# Berberine Attenuated Proliferation, Invasion and Migration by Targeting the AMPK/HNF4α/WNT5A Pathway in Gastric Carcinoma

**DOI:** 10.3389/fphar.2018.01150

**Published:** 2018-10-19

**Authors:** Qian Hu, Lingli Li, Xin Zou, Lijun Xu, Ping Yi

**Affiliations:** ^1^Institute of Integrated Traditional Chinese and Western Medicine, Tongji Hospital, Tongji Medical College, Huazhong University of Science and Technology, Wuhan, China; ^2^Department of Integrated Traditional Chinese and Western Medicine, Tongji Hospital, Tongji Medical College, Huazhong University of Science and Technology, Wuhan, China

**Keywords:** berberine, metformin, gastric cancer, HNF4α, WNT

## Abstract

**Background:** Recent epidemiologic studies have found that patients with diabetes have a higher risk of gastric cancer (GC), and the long-term use of metformin is associated with a lower risk of gastric cancer. It is believed that blocking tumor energy metabolic alterations is now emerging as a new therapeutic approach of cancer. Berberine, a natural isoquinoline alkaloid, could modulate lipid metabolism and glucose homeostasis by regulating the expression of HNF4α in many metabolic diseases. Here, we investigated the effect of Berberine on GC and its possible molecular mechanism through targeting HNF4α.

**Methods and Results:** (1) AGS and SGC7901 gastric cancer cells were treated with Berberine (BBR). We found that in AGS and SGC7901 cell, BBR inhibited cell proliferation in a time- and dose-dependent manner through downregulating *C-myc.* BBR also induced G_0_-G_1_ phase arrest with the decreased expression of cyclin D1. Moreover, BBR attenuated the migration and invasion by downregulating MMP-3. (2) The lentivirus infection was used to silence the expression of HNF4α in SGC7901 cell. The results demonstrated that the knockdown of HNF4α in SGC7901 slowed cells proliferation, induced S phase arrest and dramatically attenuated gastric cancer cells’ metastasis and invasion. (3) We performed GC cells perturbation experiments through BI6015 (an HNF4α antagonist), AICAR (an AMPK activator), Compound C (AMPK-kinase inhibitor), metformin and BBR. Our findings indicated that BBR downregulated HNF4α while upregulating p-AMPK. Moreover, the inhibition of HNF4α by BBR was AMPK dependent. (4) Then the LV-HNF4α-RNAi SGC7901 cell model was used to detect the downstream of HNF4α *in vitro*. The results showed that the knockdown of HNF4α significantly decreased WNT5A and cytoplasmic β-catenin, but increased E-cadherin *in vitro*. Berberine also downregulated WNT5A and cytoplasmic β-catenin, the same as LV-HNF4α-RNAi and BI6015 in GC cells. (5) Finally, the SGC7901 and LV-HNF4α-RNAi SGC7901 mouse-xenograft model to evaluate the effect of BBR and HNF4α gene on GC tumor growth. The result illustrated that BBR and knockdown of HNF4α suppressed tumor growth *in vivo*, and BBR decreased HNF4α, WNT5A and cytoplasmic β-catenin levels, the same effect as HNF4α knockout *in vivo*.

**Conclusion:** BBR not only had proliferation inhibition effect, attenuated the invasion and migration on GC cell lines, but also suppressed the GC tumor growth *in vivo*. The anti-gastric cancer mechanism of BBR might be involved in AMPK-HNF4α-WNT5A signaling pathway. HNF4α antagonists, such as BBR, could be a promising anti-gastric cancer treatment supplement.

## Introduction

Gastric cancer is one of the leading deadly malignancies in both sexes worldwide, especially in eastern Asia (Torre et al., 2016). In 2017, it is estimated the number of stomach cancer deaths in China will reach 2.21 million, ranking third in the world and second in China (Balakrishnan et al., 2017; Chen et al., 2018). Recent epidemiologic studies have found that patients with diabetes have a higher risk of gastric cancer, and the long-term use of metformin is associated with a lower risk of gastric cancer compared with the lack of use of metformin (Tseng and Tseng, 2014; Li et al., 2018). It is believed that the occurrence of gastric cancer is related to energy metabolism, blocking these metabolic alterations is now emerging as a new therapeutic approach of cancer, and some of metabolic enzymes involved in the glycolytic pathway are currently considered as new therapeutic targets (Song et al., 2015).

Hepatocyte nuclear factor 4α (HNF4α) is a nuclear transcription factor that binds DNA as a homodimer (Jucá et al., 2017). Multiple HNF4α isoforms exist in humans and are suggested to have different physiological roles in development and transcriptional regulation of target genes. Previous studies have found that HNF4α is a key regulator of a number of genes involved in glucose, cholesterol and fatty acid metabolism, and HNF4α mutations cause monogenic type 2 diabetes of the young (Rhee et al., 2006; Love-Gregory and Permutt, 2007). Recent studies have shown that changes in HNF4α gene expression are associated with many types of cancers, such as hepatocellular carcinoma, renal cell carcinoma, gastric adenocarcinoma, small intestine carcinoma, and colorectal cancer (Darsigny et al., 2010). Moreover, many studies have found that HNF4α is highly expressed in gastric cancer tissues, especially in Asia (Chang et al., 2015; Ma et al., 2017). The expression of HNF4α is seen in primary gastric adenocarcinomas and in metastases of gastric carcinoma to the breast, but is absent in primary breast carcinomas, and in metastases of breast carcinomas to the stomach (Van der Post et al., 2014). What’s the molecular mechanism of HNF4α, a nuclear receptor that activates the expression of genes involved in glucose, fatty acid and cholesterol metabolism, in the development of gastric cancer?

Berberine (BBR) is a natural isoquinoline alkaloid extracted from plants, such as berberis aquifolium, vulgaris, aristata, and tinospora cordifolia (Tabeshpour et al., 2017). BBR was used to be an antibacterial agent in China for a long time. Over the past decade, It has been reported that BBR could also regulate plasma lipid and glucose levels in type 2 diabetes animal models and humans (Kumar et al., 2015; Pirillo and Catapano, 2015; Tabeshpour et al., 2017; Wang H. et al., 2018). Our previous studies have also indicated that BBR can stimulate insulin secretion and modulate lipids in impaired glucose tolerance rats (Leng et al., 2004), reverse free-fatty-acid-induced insulin resistance in 3T3-L1 adipocytes (Yi et al., 2008), even attenuate intestinal mucosal barrier dysfunction and immune barrier damages in type 2 diabetic rats (Gong et al., 2017). And the molecular mechanism which BBR modulates lipid metabolism, inhibits hepatic gluconeogenesis and enhances insulin secretion is targeting HNF4α (Wang et al., 2008; Wei et al., 2016). Recent studies revealed that BBR can inhibit the growth of gastric tumor, the anti-cancer effect of BBR is through inhibiting glucose uptake and reducing the transcription of glucose metabolism relative genes (Wang et al., 2016; Mao et al., 2018). So does Berberine share same molecular target in metabolism regulation and in anti-tumor developing?

In the present study, firstly, we investigated the anti-gastric cancer effect of BBR on GC cell lines. Secondly, the lentivirus infection was used to silence HNF4α in SGC7901 cell to demonstrate whether HNF4α was a key target for anti-gastric cancer. Then, BI6015 (an HNF4α antagonist), AICAR (an AMPK activator), Compound C (AMPK-kinase inhibitor), metformin and LV-HNF4α-RNAi SGC7901 cell model was used to detect the molecular mechanism of BBR in GC cell lines. Finally, we used SGC7901 and LV-HNF4α-RNAi SGC7901 mouse-xenograft models to demonstrate the effect of BBR and HNF4α *in vivo*. Our results revealed that Berberine, a metabolism-regulated drug, could inhibit GC cells proliferation, migration and invasion and suppress GC tumor growth. The anti-gastric cancer mechanism of BBR might be involved in AMPK- HNF4α-WNT5A signaling pathway and HNF4α is a key molecule target.

## Materials and Methods

### Materials

Berberine chloride (BBR), dimethyl sulfoxide (DMSO) and Compound C were obtained from Sigma-Aldrich, United States. BI6015 (an HNF4α antagonist) and AICAR (AMPK activator) were provided by the TOCRIS Bio-Techne. Metformin was purchased from Tongji Hospital. Cultrex PathClear Basement Membrane Extract (BME) was obtained from RDsystems. Mouse monoclonal Antibodies against human AMPK, p-AMPK, HNF4α, β-catenin, E-cadherin, cyclin D1, MMP-3, and C-myc were obtained from Cell Signaling Technologies (Boston, MA, United States), WNT5A was purchased from Abgent. Another Antibody against HNF4α (sc-374229) was obtained from Santa Cru Biotechnology (Santa Cruz, CA, United States), which was used for immunohistochemistry of mouse tumor tissue.

### Cell Culture

Hubei Biossci Biological Co., Ltd., supported the GC cell lines SGC7901 and AGS. The SGC7901 and AGS cells were cultured in RPMI 1640 medium (HyClone, China) with 10% fetal bovine serum (FBS) (SiJiQing, China), 1% penicillin-streptomycin-amphotericin solution at 37°C in a humidified chamber with 5% CO_2_. When cells were in logarithmic growth phase, they were given drug treatment.

### Small Interfering RNA Transfection

Lentiviruses which carried the small interfering human HNF4α RNA (siRNA) were purchased from Gikai Gene and used to transfect cell to silence the expression of HNF4α. Lentiviruses without the siRNA gene were generated as a control and designated as Lenti-GFP. The transfection medium was removed after 12 h, replaced with fresh medium, and the cells then grown in 5% CO_2_ at 37°C for an additional 48–72 h. Stably transfected cells were selected by puromycin. RT-PCR and western blot analyses were performed to confirm target knockdown by siRNA.

### MTT Assay

Cell growth was assessed with a standard 3-(4,5-dimethyl-2-tetrazolyl)-2,5-diphenyl-2H tetrazolium bromide (MTT) assay. A total of 5 × 10^3^ cells were seeded in each well of 96-well culture plates. After 8 h, the cells were treated with different concentrations of drugs (Berberine 10∼80 μM, metformin 10∼80 mM). After 24, 48, and 72 h, the culture medium was removed, and 20 μl of a 0.5 mg/ml solution of MTT (Sigma-Aldrich) was added to each well. The plates were then incubated for 4 h at 37°C. The MTT solution was then removed and replaced with 100 μl of DMSO (Wuhan Goodbio Technology Co., Ltd.) per well, and the absorbance at 570 nm was measured using an Enzyme-linked immunoassay (Synergr2 BioTek, United States).

### Migration and Invasion Assays

Cells were trypsinized and resuspended in serum-free medium at a concentration of 1 × 10^5^ cells/ml. Approximate 2 × 10^4^ cells were seeded in the upper chamber (Millipore, United States) seated on 24-well plate, and 750 μl complete medium with 10% FBS was added to each well of the plate as the lower chamber. After 16 h of incubation, non-filtered cells were removed using a cotton swab. Migratory cells on the underside of membrane were fixed and stained with 0.1% crystal in five different fields at 200× magnification.

Cell invasion was examined using Corning chamber assays. The bottom of the Transwell membrane was pretreated with 100 μl Cultrex PathClear Basement Membrane Extract (RDsystems) for 2 h at 37°C. Afterward, 5 × 10^4^ cells re-suspended in serum-free 1640 were plated in the upper chamber of a trans-well apparatus (8.0 um pore, Corning), while 750 μl of 1640 with 10% FBS were provided in the lower chamber. After incubation at 37°C for 24 h, cells that migrated to the bottom of the membrane were attached and fixed, stained with 0.5% crystal violet, and cells in the upper chamber were removed with a cotton sticker. The images were acquired using a light microscope. The cells were examined under a fluorescence microscope. (×200, Nikon).

### Cell Cycle Analysis by Flow Cytometry

Cell cycle distribution assay by flow cytometry. Cell cycle analysis was determined by PI staining. Briefly, AGS and SGC7901 cells were treated with drugs for 48 h, and then were blended with 13-methyl-palmatrubine. Treated and control cells were harvested, washed twice with phosphate buffered saline (PBS) and fixed with pre-cooled 70% ethanol for 24 h at 4°C. Fixed cells were washed, pelleted, re-suspended in 500 μl PBS containing 50 μg RNase A at 37°C and then stained with 5 μg PI in the dark at room temperature for 30 min. Finally, cell cycle distributions were immediately assessed using a FACSort Flow Cytometer (BD Biosciences, San Jose, CA, United States).

### Western Blot Analysis

Cells were washed twice with cold PBS and then were suspended in 100 μl RIPA lysis buffer for 30 min at 4°C, vortexed every 10 min, and then centrifuged at 12000 ×*g* for 15 min. The supernatants containing the total protein extracts were collected. Protein concentration was measured by the BCA. Sample proteins (60 μg of protein/lane) on a 10% SDS-polyacrylamide electrophoresis gel (SDS–PAGE).The electrophoresis was carried out first at 80 V for 30 min and followed by 120 V for 60 min. The proteins were separated using SDS–PAGE gel and transferred onto NC membranes (0.4 um, Millipore, United States). The transferred NC membranes were incubated for 1 h with 5% non-fat milk blocking buffer, the primary antibody (1:800 or 1:1000) were incubated overnight with gentle agitation at 4°C. The membranes were washed three times and incubated with the second antibody (1:8000 or 1:10000) at room temperature for 1 h and subsequently were visualized with a near-infrared double color laser imaging system (Odyssey, Lincoln, NE, United States).

### RNA Isolation and Quantitative PCR Analyses

Total RNA was extracted from cultured cells in the exponential phase of growth using the TRIzol Reagent (Magen, Wuhan) according to the manufacturer’s instructions. cDNA was synthesized from 2 μg of total RNA using the 5X All-In-One RT MasterMix (abm) at 42°C for 15 min and at 85°C for 5 min. Real-time PCR reactions were performed using EvaGreen 2X qPCR MasterMix at 95°C for 10 min, 95°C for 15 s and 60°C for 60 s, 40 cycles. Relative quantity of HNF4α, WNT5A, C-myc, CyclinD1, β-catenin, MMP-3 and E-cadherin were calculated using the ΔΔCt method with GAPDH as reference control. The reproducibility of the measurements was assessed by performing triplicate reactions. The primer sequences are listed in **Table 1**.

**Table 1:** Primers for RT-PCR assay.

Gene	Forward (5′-3′)	Reverse (5′-3′)
AMPKα 2	CAATCGTTCTGTCGCCACTC	TCTTTCACAGCCTCATC ATCAA
HNF4α	TGGTGGACAAAGACAAG AGGAA	GAGCGCATTGATGGAGGG
WNT5a	CGACTATGGCTACCGCTTTG	AGGGCATCACCCACCTTG
β-catenin	TGCTGAAGGTGCTATCT GTCTG	CCTTCCATCCCTTCC TGTTT
CyclinDl	GTGACCACCACCCCAACAA	CCTTTCCCGACCCTGCTA
E-cadherin	CCGCCGGCGTCTGTAGGAA	AGGGCTCTTTGACCACCG CTCTC
MMP-3	GGCCTGGAACAGTCTTGGC	TGTCCATCGTTCATCAT CGTCA
C-MYC	GGATTCTCTGCTCTCC TCGAC	CTCCAGCAGAAGGTG ATCCA
GAPDH	AATCCCATCACCATCT TCCAG	GAGCCCCAGCCTTC TCCAT

### Xenograft Model Analysis

Animal experiments were carried out according to the institutional guidelines and regulations. All animal studies were approved by the Huazhong University of Science and Technology Institutional Animal Care and Use Committee (S787). We purchased 18 female athymic mice (BALB/c-nude; 4 weeks old; 13–16 g) from Hunan Slake Jingda Experimental Co., Ltd. (Hunan, China). The animals were maintained under specific pathogen-free condition using a laminar airflow rack and had continuous free access to sterilized flood and autoclaved water. Nine mice were inoculated with SGC7901 cells (10^7^ cells per animal) subcutaneously on the right flank regions of the mouse. The rest 9 mice were inoculated with normal control (*n* = 3), Lenti-GFP (*n* = 3), and LV-HNF4α-RNAi (*n* = 3) SGC7901 cells (10^7^ cells per animal), respectively, subcutaneously on the right flank regions of the mouse. Seventy-two hours later, the xenografts were identifiable as a mass of more than 6 mm in maximal diameter in all recipients. The SGC7901 mouse-xenograft models were randomly assigned to three groups (control group, *n* = 3; BBR group, *n* = 3; and MET group, *n* = 3). Mice were gavaged with PBS alone (control), BBR (100 mg/kg/day), MET (250 mg/kg/day) every other day starting on day 3. Tumor volume was calculated every third day as follows: tumor volume (mm^3^) = [tumor length (mm) × tumor width (mm)^2^]/2. All animals were sacrificed on day 18 after treatment. All animals were alive during the observation.

### Immunohistochemistry Staining

Solid tumors were removed from sacrificed mice and fixed with 4% formaldehyde. Paraffin-embedded tumors tissue were sliced on 4-μm thick and mounted on APES-coated slides. Slides were deparaffinized in xylene and rehydrated in graded ethanol. Endogenous peroxidase activity was quenched with a 3% hydrogen peroxide solution in methanol at room temperature for 30 min, followed by rinsing in pH 6.0 PBS. After antigen retrieval in a water bash set in a 10 mmol/L citrate buffer (pH 6.0) at 94°C for 8 and 10 min, respectively, the slides were immediately cooled for 20 min at room temperature, Non-specific binding sites were blocked by incubation with wash buffer containing 10% normal goat serum at 37°C for 30 min. The sections were then incubated overnight at 4°C with a primary antibodies against HNF4α (at 1:100; Santa Cruz), WNT5A (at 1:80; abs113167 Abgent), and β-catenin (at 1:100; Cell Signaling Technologies). The positive stains were shown as brown color by peroxidase substrate solution DAB, and samples were lightly counterstained with hematoxylin. Specimens were examined under a light microscope.

### Statistical Analysis

All data were analyzed by Graph Pad Prism 6 software and expressed as the mean ± SD. Significance among groups was analyzed by one-way analysis of variance (ANOVA), followed by Dunnett’s multiple comparison post-test. A value with P < 0.05 was considered significant.

## Results

### Effect of Berberine on Cell Proliferation in GC Cells

**FIGURE 1:**
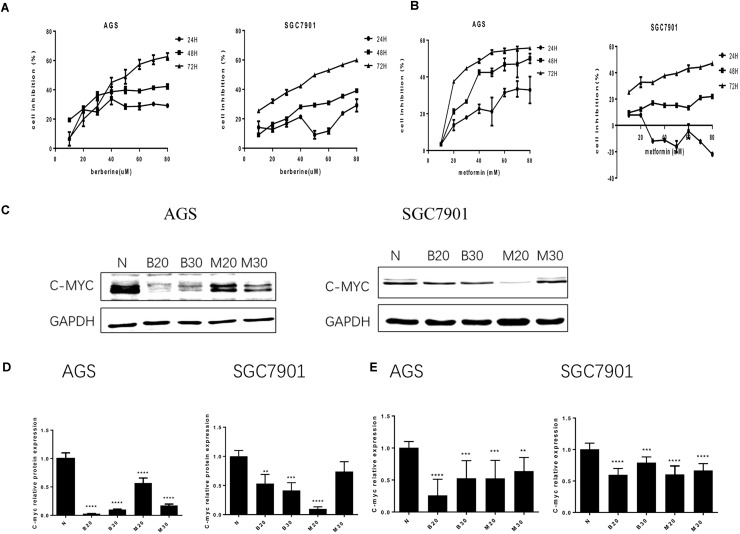
Effect of Berberine on proliferation in GC cells. Human gastric cancer AGS and SGC7901 cells were treated with BBR and metformin. **(A)** Effect of BBR on cell inhibition at 24, 48, and 72 h. **(B)** Effect of MET on cell inhibition at 24, 48, and 72 h. **(C)** The protein expression of *C-myc* in AGS and SGC7901 cells 48 h after treatment. **(D)** The quantification of *C-myc* protein levels in AGS and SGC7901 cells. **(E)** The quantification of mRNA expression of *C-myc* in AGS and SGC7901 cells 48 h after treatment. Data were presented as the means ± SD. N, untreated cells (normal control group); B20, Berberine (20 μM); B30, Berberine (30 μM); M20, Metformin (20 μM); M30, Metformin (30 μM); *n* = 6 for MTT and *n* = 3 for western-blot and RT-PCR. ^∗∗^*P* < 0.01, ^∗∗∗^*P* < 0.001, ^∗∗∗∗^*P* < 0.0001 vs. normal control group.

To address the effect of BBR on gastric cancer cells growth, we evaluated GC cell viability using MTT assay. As shown in **Figure 1A**, treatment with increasing concentration of BBR (10–80 μM) resulted in growth inhibition of AGS and SGC7901 cells, also in a time-dependent manner (24, 48, and 72 h), although BBR had an unstable inhibitory effect on SGC7901 cells at 24 h (40–60 μM). Metformin (MET, 10–80 mM) was chosen as positive control (Chang et al., 2015). The result showed that MET had same proliferation inhibition effect on AGS and SGC7901, with a time- and dose-dependent manner, expect for SGC7901 cells at 24 h (**Figure 1B**). Consider reducing the toxicity of drugs to cells and maintaining certain inhibitions against cancer cells, 20 and 30% inhibitory concentration of BBR and MET at 48 h were chosen for the follow-up experiment. The proto-oncogene *C-myc* is closely related to the proliferation of cancer cells. We detected the changes of proliferative gene *C-myc* after treating with BBR and MET by western blotting and RT-PCR. As shown in **Figures 1C,D**, BBR (20 and 30 μM) and MET (30 mM) showed the best inhibitory effect on proto-oncogene *C-myc* in AGS cells (*P* < 0.001). In SGC7901 cells, MET (20 mM) showed a best inhibition (*P* < 0.001). On the whole, treatment with BBR and MET obviously reduced the protein levels of proliferative gene *C-myc*, compared with untreated AGS and SGC7901 cells. As showed in **Figure 1E**, the results showed the same inhibiting trend in mRNA levels after treating with BBR and MET. What’s more, the low concentration of BBR and MET had a better effect on inhibiting mRNA expression of proliferative gene *C-myc* than high concentration in both AGS and SGC7901 cells (*P* < 0.01).

### Berberine Induced G_0_/G_1_ Phase Cell Cycle Arrest

**FIGURE 2:**
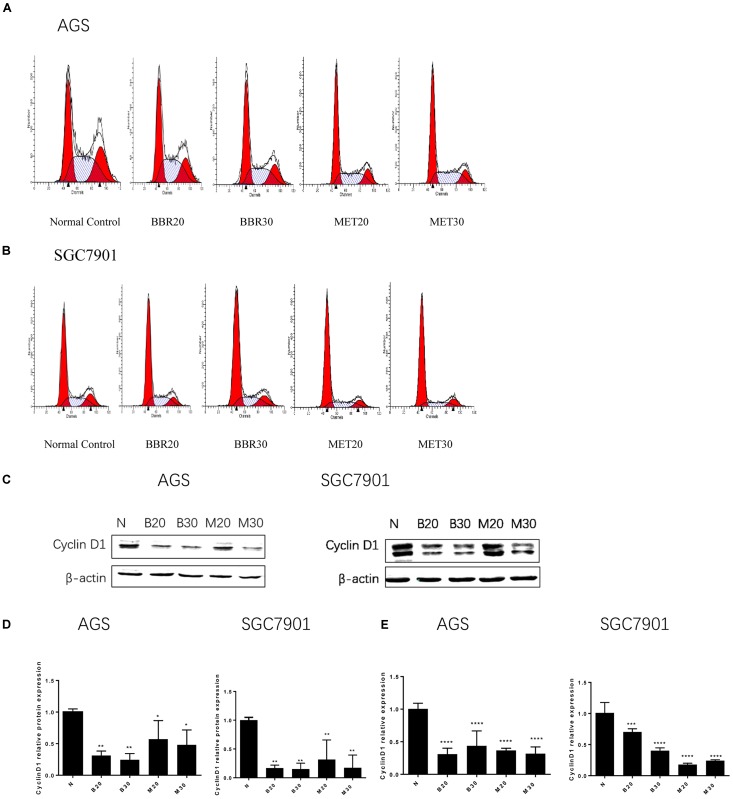
Berberine induced G0/G1 phase cell cycle arrest. Human gastric cancer AGS and SGC7901 cells were treated with BBR and MET. At 48 h after treatment, the numbers of cells in G_o_–G_1_, S, and G_2_-M phase were calculated by flow cytometry. **(A)** AGS cells. **(B)** SGC7901 cells. **(C)** The protein expression of Cyclin D1 in AGS and SGC7901 cells 48 h after treatment. **(D)** The quantified western blot of Cyclin D1 in AGS and SGC7901 cells. **(E)** At 48 h after treatment, the quantified mRNA expression of Cyclin D1 in AGS and SGC7901 cells. Data were presented as the means ± SD. *n* = 3 for western-blot and RT-PCR. ^∗^*P* < 0.05, ^∗∗^*P* < 0.01, ^∗∗∗^*P* < 0.001, ^∗∗∗∗^*P* < 0.0001 vs. Normal control group.

To investigate how BBR influences cancer cell viability, we stained AGS and SGC7901 with PI and quantified the number of cells in G_0_-G_1_, S, G_2_-M period by flow cytometry. We found that the number of cells in G_0_-G_1_ period increased in both AGS and SGC7901 cells after treating with BBR and MET (*P* < 0.05, *P* < 0.01, and *P* < 0.001) (**Figures 2A,B** and **Tables 2**, **3**). Cyclin D1 is a key protein implicated in the transition of the G_0_-G_1_ phase, a decline in Cyclin D1 level indicates a G_0_-G_1_ arrest in cells. We examined both the protein and mRNA levels of Cyclin D1 in AGS and SGC7901 cells separately after the treatment of BBR and MET. The results showed both BBR and MET had negative influence on the protein expression of Cyclin D1 in AGS and SGC7901 cells, and there was a dose-dependent inhibition (*P* < 0.05 and *P* < 0.01) (**Figures 2C,D**). As shown in **Figure 2E**, BBR and MET also downregulated the mRNA level of Cyclin D1 in both AGS and SGC7901 cells, which was consistent with the protein results (*P* < 0.001).

**Table 2:** Cell cycle ratios of BBR on AGS cell (mean + SD) (%).

Group	G0-G1	S	G2-M
Control	42.590 + 5.037	40.728 + 4.758	18.170 + 4.341
Berberine (20 umol/L)	44.145 + 3.856^∗∗∗^	38.118 + 1.223	17.735 + 2.673
Berberine (30 umol/L)	46.720 + 4.365^∗∗∗^	36.982 + 2.727^∗^	16.302 + 3.071
Metformin (20 mmol/L)	47.798 + 2.224^∗∗∗^	36.217 + 1.052^∗^	15.972 + 2.156
Metformin (30 mmol/L)	47.058 + 2.788^∗∗∗^	36.210 + 1.776^∗^	16.733 + 4.457

*n = 4 for cell cycle analysis vs. Control, ^∗^P < 0.05 and ^∗∗∗^P < 0.001.*

**Table 3:** Cell cycle ratios of BBR on SGC7901 cell (mean + SD) (%).

Groups	G0-G1	S	G2-M
Control	58.757 + 0.379	25.960 + 1.200	15.283 + 1.424
Berberine (20 umol/L)	63.533 + 0.973^∗^	24.557 + 1.236	11.913 + 1.415^∗^
Berberine (30 umol/L)	65.050 + 1.131^∗∗^	22.403 + 1.297^∗^	12.550 + 0.650
Metformin (20 mmol/L)	69.020 + 1.750^∗∗∗^	21.283 + 2.182^∗^	9.663 + 0.686^∗∗^
Metformin (30 mmol/L)	69.370 + 4.038^∗∗∗^	18.880 + 2.252^∗∗^	11.753 + 2.449^∗^

*n = 3 for cell cycle analysis vs. Control, ^∗^P < 0.05, ^∗∗^P < 0.01, and ^∗∗∗^P < 0.001.*

### Berberine Inhibited the Migration and Invasion in GC Cells

**FIGURE 3:**
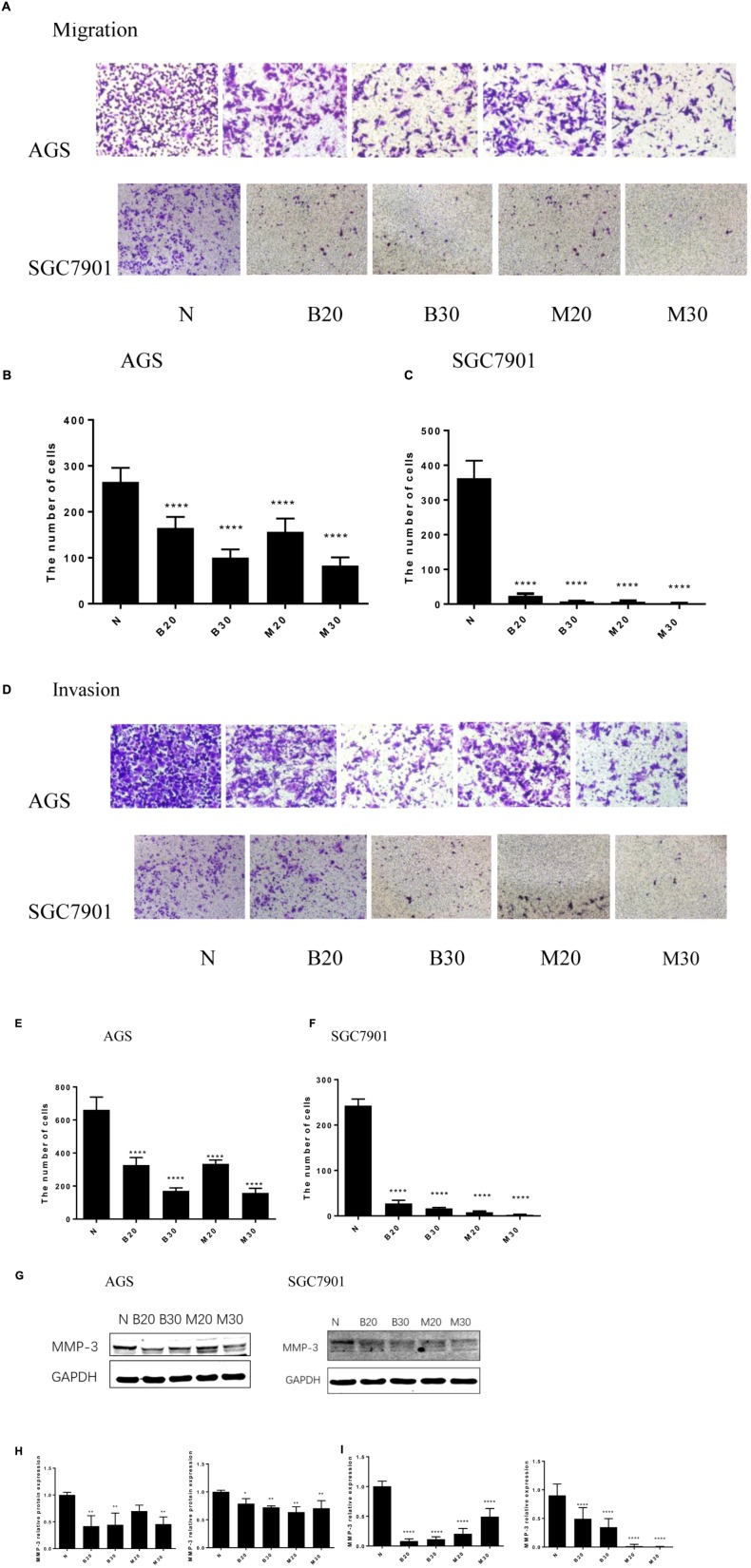
Berberine inhibited the migration and invasion of GC cells. Human gastric cancer AGS and SGC7901 cells were treated with BBR and MET. Effect of BBR and MET on migration of GC cells after 48 h treatment. Representative images from experiment **(A)** and quantification of migration cell number (**B**: AGS cells; **C**: SGC7901 cells). Effect of BBR and MET on invasion of GC cells after 48 h treatment. Representative images from experiment **(D)** and quantification of invasion cell number (**E**: AGS cells; **F**: SGC7901 cells). Data were presented as the means ± SD, and representative data from at least three independent experiment are shown. **(G)** Protein expression of MMP-3 in AGS and SGC7901 cells 48 h after BBR and MET treatment. **(H)** The quantified western blot of MMP3 in AGS and SGC7901 cells. **(I)** Quantified mRNA expression of MMP3 in AGS and SGC7901 cells 48 h after BBR and MET treatment. *n* = 3 for western-blot and RT-PCR. ^∗^*P* < 0.05, ^∗∗^*P* < 0.01, ^∗∗∗∗^*P* < 0.0001 vs. Normal control group.

Tumor cell invasion and metastasis are the main characteristics of malignant tumor, which are the leading causes of death in patients with malignant tumor. We used the Transwell assay to examine the effect of BBR and MET on GC cells migratory and invasion. As shown in **Figures 3A–C**, the migration numbers of AGS and SGC7901 cells significantly reduced through the Transwell chamber microspores in the treatment group (*P* < 0.001). When chamber was coated with Matrigel, which was used to mimic the extracellular matrix (ECM) around tumors and to detect the invasion ability of cells. As shown in **Figures 3D–F**, The BBR-treated and metformin-treated AGS cells had a significant lower invasion than the untreated cell, the same effect was observed in SGC7901 cells (*P* < 0.001). With the increase concentration of BBR and MET, the number of gastric cancer cells that invaded and metastasized gradually decreased. Matrix metalloproteinase-3 (MMP-3), known as inducers of EMT, is one of the several MMPs that regulate angiogenesis, invasion and metastasis (Huang et al., 2016; Chu et al., 2018; Simi et al., 2018). It was reported that MMP-3 expression level was negatively correlated with GC development (Xu et al., 2016). We used western-blot and RT-PCR to determine whether BBR can affect the expression of matrix metalloproteinase-3 (MMP-3). The results showed that BBR (20 and 30 μM) and MET (30 mM) had stable inhibitory effect on MMP-3 protein expression in AGS and SGC7901 cells (*P* < 0.01), MET (20 μM) downregulated the expression of MMP-3 in SGC7901 cells, but had no obvious inhibition in AGS cells (**Figures 3G,H**). As shown in **Figure 3I**, there was a obviously decrease in the mRNA levels of MMP-3 in BBR group compared to untreated group (N) in AGS and SGC7901 cells, MET had similar effect on AGS and SGC7901 cells (*P* < 0.01 and *P* < 0.001).

### Knockdown of HNF4α Attenuated Proliferation, Invasion, and Migration in SGC7901 Cell

**FIGURE 4:**
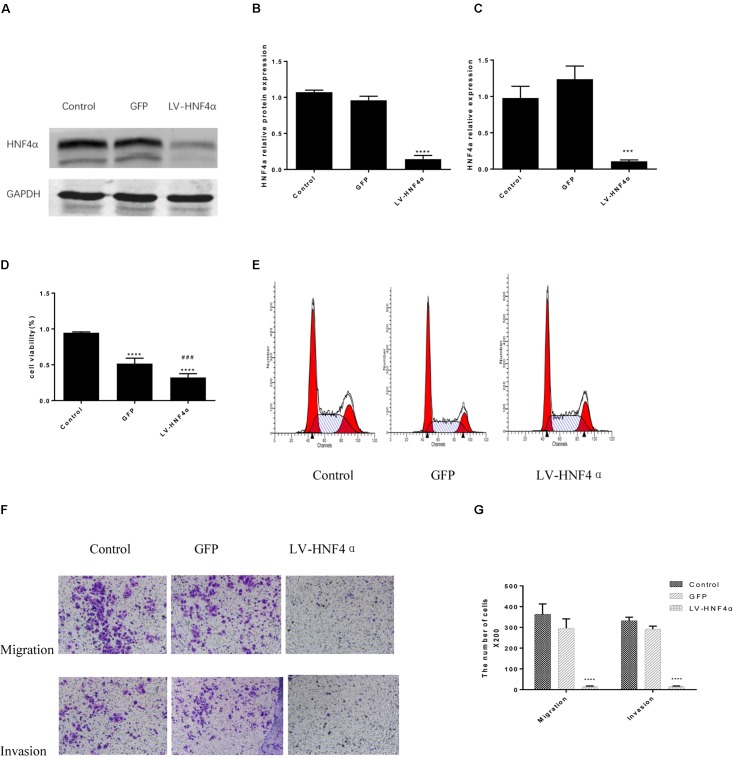
Knockdown of HNF4α attenuated proliferation, migration and invasion of SGC7901 cells. Lentivirus-mediated HNF4α siRNA transcript and empty vectors were infected into SGC7901 gastric cancer cells as the Control group and the GFP group respectively. Western-blotting and RT-PCR were used to confirm the knockdown of HNF4α and quantified. **(A)** Representative western-blot images. **(B)** Quantification of Western blot results. **(C)** Quantification of RT-PCR (*n* = 3). We examined the effect of HNF4α knockout on SGC7901 cells. **(D)** Cell proliferation (*n* = 3). **(E)** Cell cycle (*n* = 3). **(F)** Representative images of migration and invasion. **(G)** Quantification of migration and invasion cell number. Data were presented as the means ± SD, with representative data from at least three independent experiments. ^∗∗∗^*P* < 0.05, ^∗∗∗∗^*P* < 0.001 vs. Control group. ^###^*P* < 0.001 vs. GFP group.

Having observed the effect of BBR on gastric cancer, next, we explored the molecular mechanism of BBR against gastric cancer. HNF4α has been reported to be related to malignant tumor formation and metastasis (Xiang et al., 2015; Kato et al., 2018; Maan et al., unpublished). We used lentivirus-mediated transduction of SiRNA-HNF4α to silence the expression of HNF4α in SGC7901 cell, RT-PCR and western blot analyses were performed to confirm target gene knockdown by siRNA. The results showed a 10-fold downregulation of HNF4α expression (**Figures 4A–C**).

MTT assay was added to detect the proliferation in normal control cells (Control), empty vector control cells (GFP) cells and LV-HNF4α-SGC7901 cells. As shown in **Figure 4D**, comparing with the Control and GFP, the knockdown of HNF4α in SGC7901 cells significantly reduced the proliferation (*P* < 0.0001 and *P* < 0.001). Also, we stained normal control cells, GFP and LV-HNF4α-SGC7901 cells with PI and quantified the number of cells in G_0_-G_1_, S, G_2_-M period by flow cytometry. As shown in **Figure 4E** and **Table 4**, we found LV-HNF4α-SGC7901 cells increased S population compared with normal control cells and GFP cells (*P* < 0.05). Moreover, a Transwell assay was used to detect the role of HNF4α in gastric SGC7901 cell migration and invasion. The results showed that knockdown of HNF4α dramatically attenuated the migration and invasion of SGC7901 cell compared with the control cells (*P* < 0.001 and *P* < 0.001) (**Figures 4F,G**).

**Table 4:** Cell cycle ratios (mean + SD) (%).

Groups	G_0_-G_1_	S	G_2_-M
Normal control	43.68 + 0.383	35.51 + 2.210	20.807 + 1.835
GFP	48. 717 + 2.441^∗^	35.760 + 3.080	15.527 + 0.870^∗^
LV-HNF4α	40.920 + 0.934^∗∗^	40.877 + 1. 160^∗^	18.203 + 0.227

### Berberine Regulated HNF4α by Activating AMPK in GC Cells

A research has found metformin, a widely used antidiabetic drug, has antitumor activity through modulated HNF4α (Chang et al., 2015). Recent study has shown BBR modulates lipid metabolism and inhibits hepatic gluconeogenesis by decreasing expression of HNF4α in type 2 diabetic mouse (Wei et al., 2016). Our previous study illustrated BBR prevented fructose-induced insulin resistance by promoting the expression HNF4α in rat livers (Wei et al., 2012). In this study, knockdown of HNF4α in SGC7901 cell line slowed its proliferation, induced S phase arrest, dramatically attenuated gastric cancer cells metastasis and invasion and suppressed the tumor growth *in vivo*, the same anti-gastric effect as Berberine. Therefore, we examined how HNF4α was regulated by BBR in GC cell line. BI6015 (an HNF4α antagonist) and AICAR (an AMPK activator) were used and served as positive control.

**FIGURE 5:**
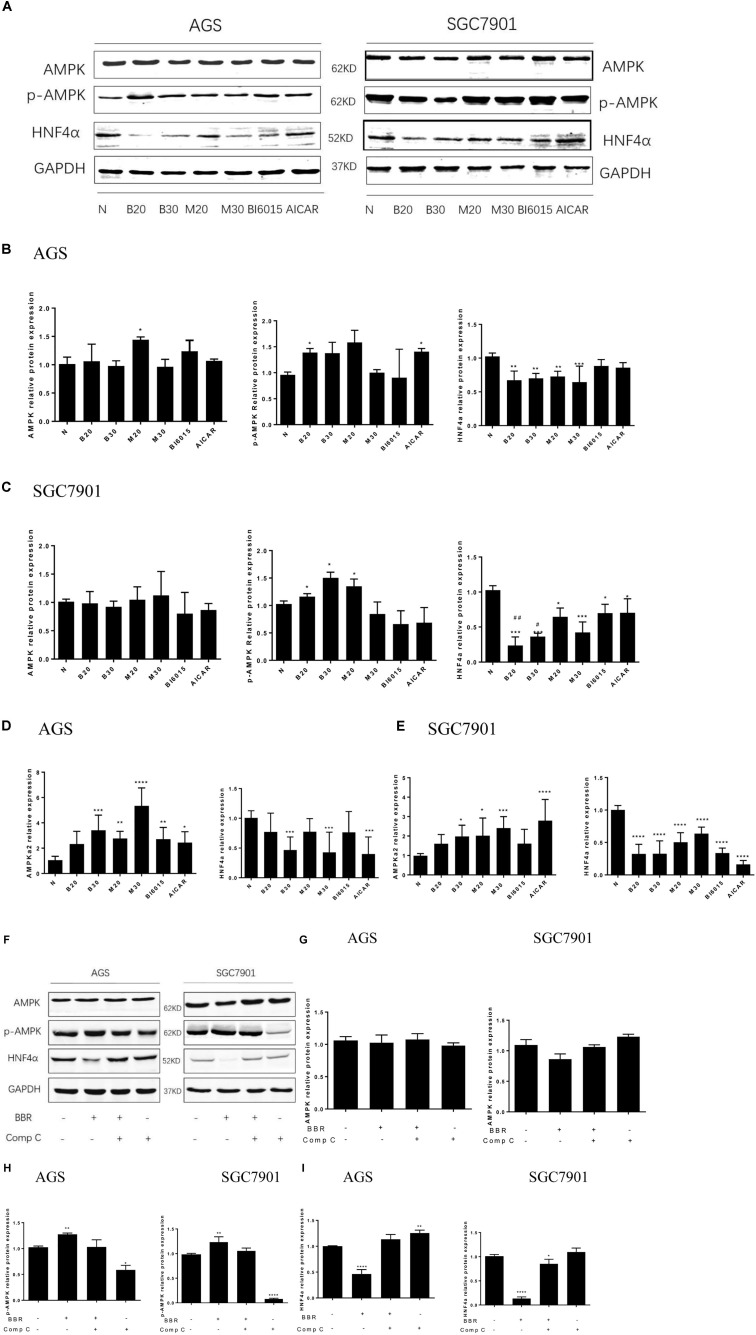
Berberine regulated HNF4α by activating AMPK in GC cells. Two GC cell lines, AGS and SGC7901, were treated with BBR (20 μM and 30 μM), metformin (20 mM and 30 mM), BI6015 (an HNF4α antagonist) and AICAR (AMPK agonist). **(A)** The protein expressions of AMPK, p-AMPK and HNF4α in AGS and SGC7901 cells 48 h after treatment. **(B,C)** The quantification of western blot in AGS cells **(B)** and SGC7901 cells **(C)**. **(D,E)** The quantified mRNA expression of AMPK and HNF4α in AGS and SGC7901 cells 48 h after treatment. Two GC cell lines, AGS and SGC7901 cells, were treated with BBR (30 μM) for 24 h, and then with compound C (20 μM) or DMSO for another 24 h. **(F)** The protein expression of AMPK, p-AMPK and HNF4α in AGS and SGC7901 cells after treatment. **(G)** The quantified western blot of AMPK in AGS and SGC7901 cells. **(H)** The quantified western blot of p-AMPK in AGS and SGC7901 cells. **(I)** The quantified western blot of HNF4α in AGS and SGC7901 cells. Data were presented as the means ± SD. *n* = 3 for western-blot and RT-PCR. *T*-test, ^∗^*P* < 0.05, ^∗∗^*P* < 0.01, ^∗∗∗^*P* < 0.001, ^∗∗∗∗^*P* < 0.0001 vs. Normal control group. ^#^*P* < 0.05, ^##^*P* < 0.05 vs. BI6015 group.

In AGS cell, BBR and MET significantly decreased the protein level of HNF4α (**Figures 5A,B**). BBR (30 μM), MET (30 mM), and AICAR reduced the mRNA expression of HNF4α (**Figure 5D**). Although there was no obvious decrease in the protein and mRNA levels of HNF4α after BI6015 treatment (**Figures 5B,D**). However, in SGC7901 cell, BI6015 decreased the protein and mRNA expression of HNF4α, the same trend in BBR treatment (**Figures 5C,E**). More thrillingly, the protein levels of HNF4α in BBR group were lower than in BI6015 group (*P* < 0.05). As we expected, MET and AICAR also decreased the protein expression of HNF4α in SGC7901 cells (**Figure 5C**).

As for the expression of p-AMPK and AMPK, we used Metformin and AICAR (an AMPK activator) as the positive control, the results showed that BBR (20 μM) increased the protein level of p-AMPK in both AGS and SGC7901 cells (*P* < 0.05), BBR (30 μM) only upregulated the protein expression of p-AMPK in SGC7901 cell (**Figures 5B,C**). Also, we observed that BBR (30 μM) group increased the mRNA level of p-AMPK in both AGS and SGC7901 cells, but not BBR (20 μM) group (**Figures 5D,E**). Although no statistically significant differences were found in the total level of AMPK after BBR-treated (**Figures 5A–E**).

To explore the interaction between AMPK and HNF4α in gastric cells, we used BBR and Compound C (AMPK inhibitor) to treat two GC cell lines and detected the changes in the protein levels of HNF4α. The results showed, BBR treatment increased p-AMPK expression and decreased HNF4α expression in GC cells, Compound C decreased p-AMPK expression and increased HNF4α expression in GC cells, there was a negative relationship between AMPK and HNF4α in GC cells (**Figures 5F,H,I**). Compound C weakened the inhibition of HNF4α by BBR, suggesting that HNF4α may be the downstream of AMPK in GC cells. Both BBR and Compound C had no effect on the total AMPK protein level (**Figure 5G**).

### Berberine Targeted the WNT/β-Catenin Signaling Through Regulating HNF4α in GC Cells

**FIGURE 6:**
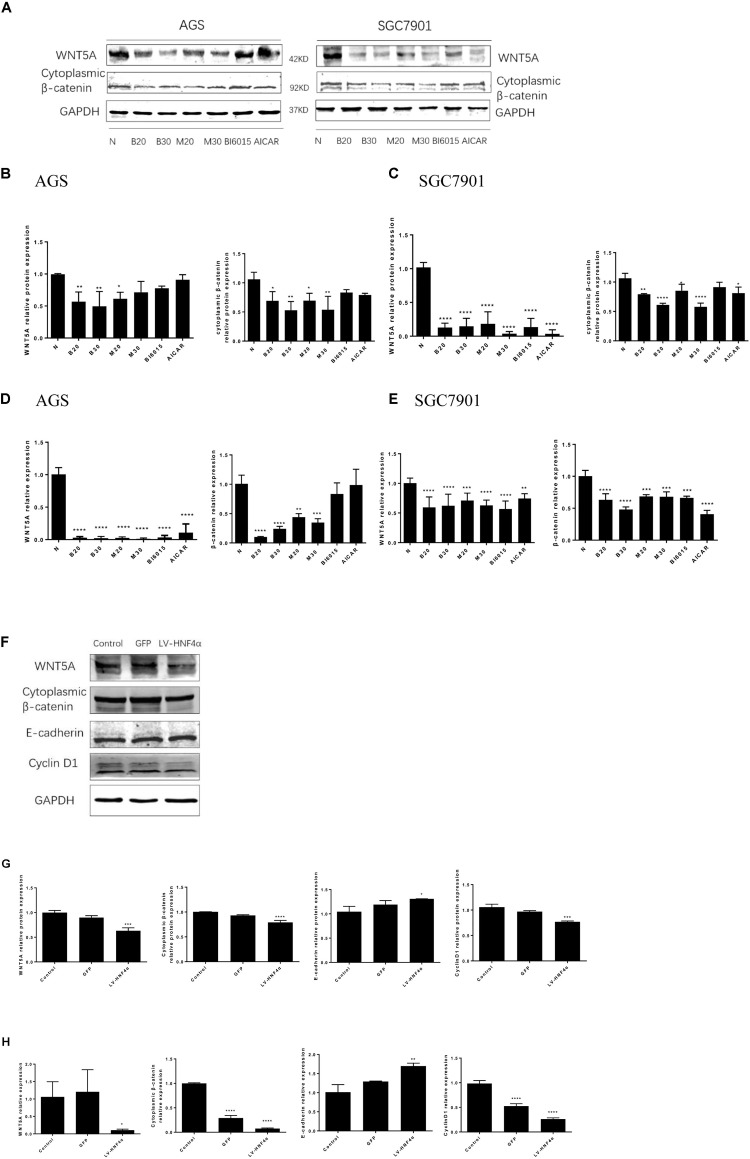
Berberine targeted WNT/β -catenin signaling through HNF4α in GC cells. First, two GC cell lines, AGS and SGC7901, were treated with BBR (20 μM and 30 μM), metformin (20 mM and 30 mM), BI6015 (an HNF4α antagonist) and AICAR (AMPK agonist). **(A)** The protein expression of WNT5A and cytoplasmic β-catenin in AGS and SGC7901 cells 48 h after treatment. **(B,C)** The quantified western blot of WNT5A and cytoplasmic β-catenin in AGS and SGC7901 cells. **(D,E)** The quantified mRNA expression of WNT5A and cytoplasmic β-catenin in AGS and SGC7901 cells 48 h after treatment. Next, an siRNA-HNF4α SGC7901 cell model was used to detect the WNT pathway. **(F)** The protein level of WNT5A, cytoplasmic β-catenin, E-cadherin and Cyclin D1 in Control, GFP and LV-HNF4α SGC7901 cell lines. **(G)** The quantified western blot of WNT5A, cytoplasmic β-catenin, E-cadherin and Cyclin D1. **(H)** The quantified mRNA level of WNT5A, cytoplasmic β-catenin, E-cadherin and Cyclin D1 in Control, GFP and LV-HNF4α SGC7901 cell lines. Data were presented as the means ± SD. *n* = 3 for western-blot and RT-PCR. ^∗^*P* < 0.05, ^∗∗^*P* < 0.01, ^∗∗∗^*P* < 0.001, ^∗∗∗∗^*P* < 0.0001 vs. Control group.

WNT signaling plays an important role in gastric cancer development, especially WNT5A, a member of the WNT family of secreted lipid-modified glycoproteins, was closely related to the gastric cancer invasion and metastasis (Kanzawa et al., 2013; Ara et al., 2016). Recent research has found that WNT5A is a direct target gene of HNF4α in GC (Nam et al., 2014). To explore how BBR exerts anti-tumor effect by HNF4α acting on downstream targets, Western blot and Real Time PCR were performed to detect the mRNA and protein levels of WNT signaling after BBR-treated. We used BBR, metformin, AICAR (AMPK activator) and BI6015 (an HNF4α antagonist) to interfere with two GC cells. As shown in **Figures 6A–E**, BBR and MET (20 mM) decreased the protein and mRNA expression of WNT5A and cytoplasmic β-catenin in both AGS and SGC7901 cells. MET (30 mM) produced the same inhibitory effect in modulating the WNT signaling pathway as MET (20 mM) in GC cells expect the protein expression of WNT5A in AGS cell. To further investigate the downstream of HNF4α in GC cells, we used SiRNA- HNF4α SGC7901 cell model to detect the WNT pathway. Western blot and Real Time PCR separately were used to test the mRNA and protein levels of WNT5A, cytoplasmic β-catenin, E-cadherin and CyclinD1. The WNT/β-catenin, being the canonical WNT pathway, which can induce the expression of CyclinD1 (Larue and Delmas, 2006). E-cadherin, another member of WNT pathway, is lost in some cancers, allowing cellular migration to the stroma (Kobayashi et al., 2018). As shown in **Figures 6F,G**, comparing with normal control cells and Lenti-GFP cells, the protein expression of WNT5A, cytoplasmic β-catenin, and Cyclin D1 decreased while the expression of E-cadherin increased in LV-HNF4α-SGC7901 cell. In mRNA level, there were obvious low levels of WNT5A, cytoplasmic β-catenin, and Cyclin D1 in LV-HNF4α-SGC7901 cell, and the level of E-cadherin in LV-HNF4α-SGC7901 cells was higher in normal control cells and Lenti-GFP cells (**Figure 6H**).

### Knockdown of HNF4α Suppressed Xenograft Tumor Growth *in vivo*

**FIGURE 7:**
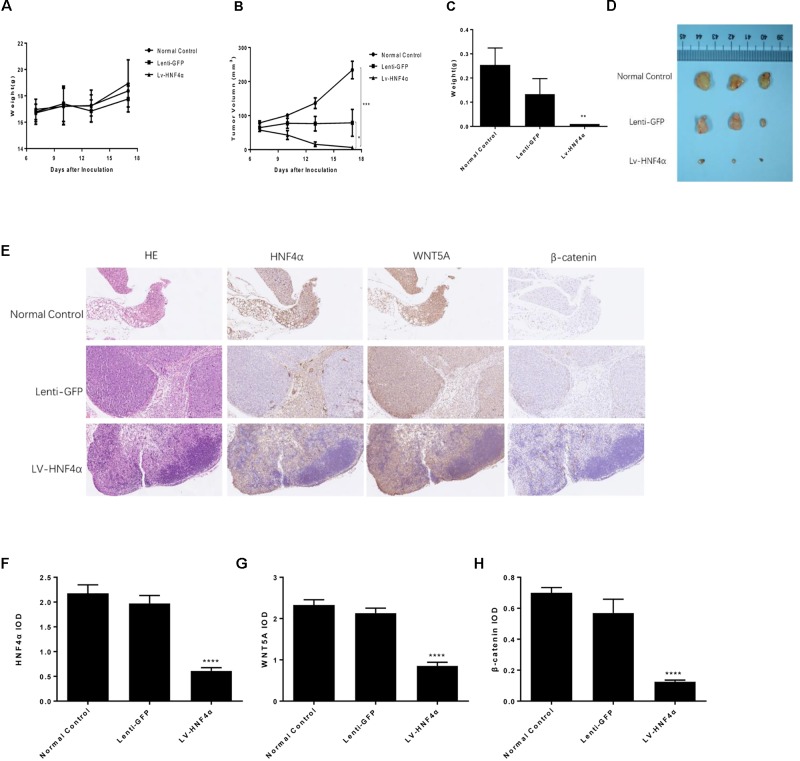
Effect of HNF4α on SGC7901 xenograft tumor growth *in vivo*. The normal control, Lenti-GFP, and LV-HNF4α SGC7901 cells were injected subcutaneously into the right scapular region of nude mice. **(A)** Body weight. **(B)** The average tumor volume is shown as the mean ± SD (mm^3^). **(C)** Tumor weight. **(D)** The tumors removed from mice are shown. **(E)** Representative images from immunohistochemical staining of HNF4α, WNT5A and β-catenin in tumors from each group (50x Lenti). **(F–H)** The quantified immunohistochemistry of HNF4α, WNT5A and β-catenin. Data were expressed as the means ± SD. ^∗^*P* < 0.05, ^∗∗^*P* < 0.01, ^∗∗∗^*P* < 0.001, ^∗∗∗∗^*P* < 0.0001 vs. normal control group.

BALB/c nude mouse models with xenografted human SGC7901 cells, Lenti-GFP and LV-HNF4α-SGC7901 cells were employed to evaluate the effect of HNF4α on tumor growth *in vivo*. Eighteen days after inoculation, the body weight remained consistent between these groups (**Figure 7A**). the average final tumor weight and volume in the LV-HNF4α group were 0.01 g and 6.268 mm^3^, respectively, which was significantly lower than those in normal control group (0.253 g, 234.0571 mm^3^) and Lenti-GFP group (0.133 g, 71.928 mm^3^) (*P* < 0.05) (**Figures 7B–D**). There was no statistical difference in tumor weight and volume between normal control group and Lenti-GFP group. Those results suggested that knockdown of HNF4α suppressed gastric cancer tumor growth *in vivo*.

We used immunohistochemistry to exam the HNF4α, WNT5A and cytoplasmic β-catenin expression in gastric cancer tumors of mice. The results showed that the expression of HNF4α decreased in LV-HNF4α group, but did not decrease in normal control group and Lenti-GFP group. The WNT5A and cytoplasmic β-catenin expression were decreased in LV- HNF4α group, which was consistent with previous cell experiments (**Figures 7E–H**) (*P* < 0.0001).

### Berberine Suppressed Xenograft Tumor Growth *in vivo*

**FIGURE 8:**
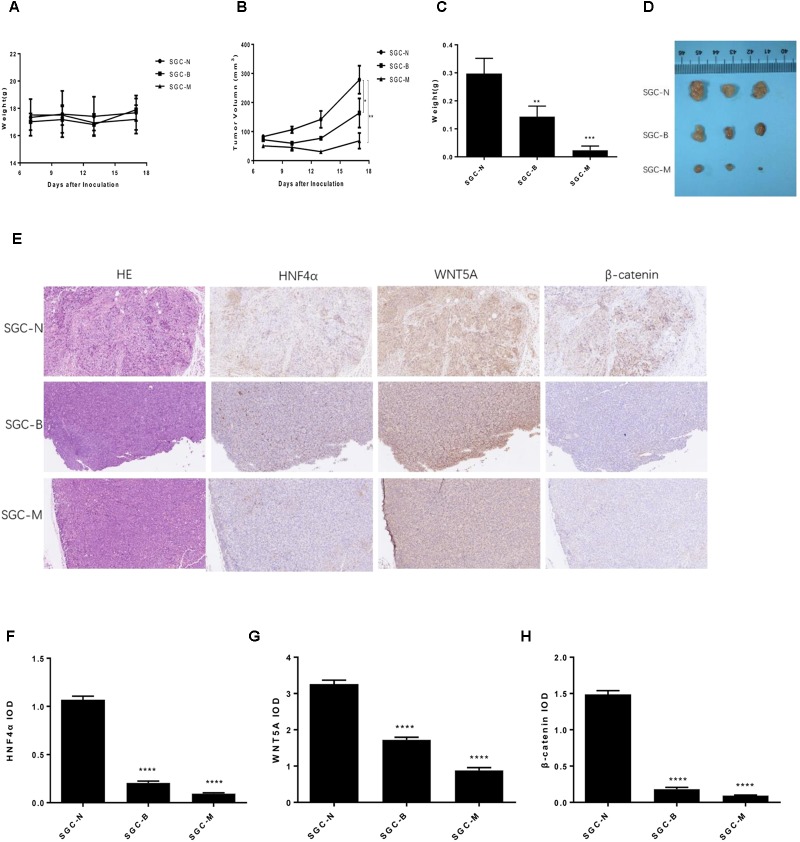
Effect of BBR on SGC7901 xenograft tumor growth *in vivo*. The SGC7901 cells were injected subcutaneously into the right scapular region of nude mice, and were given normal saline, Berberine or metformin treatment. **(A)** Body weight. **(B)** The average tumor volume is shown as the means ± SD (mm^3^). **(C)** Tumor weight. **(D)** The tumors removed from mice are shown. **(E)** Representative images from immunohistochemical staining of HNF4α, WNT5A and β-catenin in tumors from each group (50× magnification). **(F–H)** The quantified immunohistochemistry of HNF4α, WNT5A and β-catenin. Data were expressed as the means ± SD. ^∗^*P* < 0.05, ^∗∗^*P* < 0.01, ^∗∗∗^*P* < 0.001, ^∗∗∗∗^*P* < 0.0001 vs. normal group.

The SGC7901 cells were injected subcutaneously into BALB/c nude mouse, normal saline, Berberine and metformin were treated, respectively. During the 18 days treatment, the weight of each group was not statistically significant (**Figure 8A**). As shown in **Figures 8B–D**, 250 mg/kg MET (SGC-M) significantly suppressed the tumor growth *in vivo* compared with normal control group (SGC-N). The average final tumor weight and volume in the SGC-M group were 0.023 g and 68.168 mm^3^, respectively, which was significantly lower than those in SGC-N group (0.297 g, 278.213 mm^3^) (*P* < 0.001 and *P* < 0.01). There was also a reduction in tumor weight and volume in the BBR (100 mg/kg) group (SGC-B) compared with normal control group, the average final tumor weight and volume were 0.143 g and 163.955 mm^3^ (*P* < 0.01 and *P* < 0.05).

To further investigate the insight mechanism of BBR on gastric cancer tumor *in vivo*, immunohistochemistry was used to detect the HNF4α, WNT5A and cytoplasmic β-catenin expression in xenografted tumors. As shown in **Figures 8E–H**, the BBR (100 mg/kg) and MET (250 mg/kg) decreased the HNF4α, WNT5A and cytoplasmic β-catenin expression comparing with the normal control group (*P* < 0.0001). There was no difference between BBR group and MET group in HNF4α, WNT5A and cytoplasmic β-catenin expression.

## Discussion

Berberine (BBR), an isoquinoline alkaloid isolated from Berberis vulgaris, used to be an antibacterial agent in China for a long time. Over the past decade, It has been reported that BBR could also regulate plasma lipid and glucose levels in type 2 diabetes animal models and humans (Kumar et al., 2015; Pirillo and Catapano, 2015; Tabeshpour et al., 2017; Wang H. et al., 2018). Our previous studies have indicated that BBR can stimulate insulin secretion and modulate lipids in impaired glucose tolerance rats (Leng et al., 2004), reverse free-fatty-acid-induced insulin resistance in 3T3-L1 adipocytes (Yi et al., 2008), even attenuate intestinal mucosal barrier dysfunction and immune barrier damages in type 2 diabetic rats (Gong et al., 2017). In this study, we found that BBR had proliferation inhibition effect on AGS and SGC7901 gastric cancer cell lines in a time and dose-dependent manner. We also detected the proto-oncogene expression of *C-myc*, which frequently upregulates in human cancers and accelerates cell proliferation (Dang, 2012). The results showed that BBR downregulated the mRNA and protein expression of *C-myc* in both GC cell lines, suggesting BBR has potential anti-gastric cancer effect as previous researches have shown (Zhang et al., 2014; Li et al., 2016; Yang et al., 2018).

The balance between cell cycle and cell proliferation often deregulated in cancers (Gérard and Goldbeter, 2014). Then, we detected the effects of BBR on GC cell cycle. After BBR treatment, the number of cells in G_0_-G_1_ period increased, and the protein and mRNA expression of CyclinD1 decreased significantly in both AGS and SGC7901 cells. Cyclin D1 is a key protein implicated in the transition of the G_0_-G_1_ phase, which allows cells to enter the G_1_ phase of the cell cycle and promotes cell proliferation (Gérard and Goldbeter, 2014; Wang Q. et al., 2018). The decreased expression of Cyclin D1 indicates a G_0_-G_1_ arrest in cells. So the flow cytometry, western blot and RT-PCR results all showed that BBR induced G_0_-G_1_ phase arrest in AGS and SGC7901 gastric cancer cell lines.

The depth of tumor invasion and lymph node metastasis are considered as the most important prognostic predictors of gastric cancer (Deng and Liang, 2014). It is essential to explore the mechanisms of invasion and metastasis to improve the survival rate for cancer patients (Zhu et al., 2018). We used the Transwell assay to examine whether BBR affected GC cells’ migration and invasion or not. With the increased dose of BBR, the number of gastric cancer cells that invaded and metastasized dramatically decreased. MMP-3, known as inducers of EMT, is one of the several MMPs that regulate angiogenesis, invasion and metastasis (Huang et al., 2016; Chu et al., 2018; Simi et al., 2018).It was reported that MMP-3 expression level was negatively correlated with GC development (Xu et al., 2016). We also found that BBR had stable inhibitory effect on matrix metalloproteinase-3 (MMP-3) protein and mRNA expressions in AGS and SGC7901 cells, which suggested that BBR inhibited the migration and invasion to prevent GC development through downregulating MMP-3 in AGS and SGC7901 GC cells.

Previous researches reported that Metformin (MET) inhibited gastric cancer cells metastatic traits and slowed gastric tumor growth (Courtois et al., 2017; Valaee et al., 2017). In the present study, we also found that BBR, a widely used antibacterial drug, had multiple anti-tumor efficacy on AGS and SGC7901 GC cell lines as MET. We know BBR shares many features in metabolism regulation with MET. Recent researches have demonstrated that MET acted directly on cancer cells by targeting tumor metabolism, and inhibited the migration and invasion of the esophageal carcinoma cell line EC109 (Daugan et al., 2016; He et al., 2018). Among patients with T2DM, some clinical evidences have shown those who took metformin therapy have a lower risk of GC than those who didn’t (Zhou et al., 2017; Li et al., 2018). Moreover, it was reported that the anti-gastric cancer effect of metformin was related with the HNF4α inhibition through AMPK signaling (Chang et al., 2015). AMPK directly or indirectly inhibits HNF4α and its downstream HIF-1α, which is consistent with a proposed role for AMPK in suppressing the ‘Warburg effect’ anaerobic metabolism characteristic of the malignant phenotype (Kato et al., 2018).

Hepatocyte nuclear factor 4α is a nuclear receptor that activates the expression of genes involved in endoderm development and glucose, fatty acid and cholesterol metabolism (Darsigny et al., 2010). Previous studies suggested HNF4α is a potential direct or indirect target for pharmacological drugs that act on the intestinal epithelium, which is a primary site of metabolic control (Cattin et al., 2009). Recent studies have found that HNF4α is related to malignant tumor formation and metastasis (Xiang et al., 2015; Kato et al., 2018; Maan et al., unpublished). It was significantly upregulated in multidrug-resistant GC cells (Ma et al., 2017). To assess the function of HNF4α in GC cells, we used siRNA to silence the expression of HNF4α and tested the proliferation, cell cycles, migration and invasion in SGC7901 cell. We found that knockdown of HNF4α in SGC7901 cell line slowed its proliferation, induced S phase arrest and dramatically attenuated gastric cancer cells’ metastasis and invasion. It demonstrated that HNF4α was involved in the growth, invasion and metastasis capacity of gastric cancer. HNF4α gene could serve as a “proto-oncogene” in gastric cancer development.

Our previous studies have indicated that BBR modulates lipid metabolism and inhibits hepatic gluconeogenesis by targeting HNF4α in type 2 diabetic rats (Gao et al., 2008; Wang et al., 2008; Yan et al., 2008). Is the anti-gastric cancer effect of Berberine related with tumor metabolism? How is HNF4α regulated by BBR in GC cell line? We performed GC cell perturbation experiments through BI6015 (an HNF4α antagonist), AICAR (an AMPK activator), metformin, and Berberine. The results showed that BBR downregulated the expression of HNF4α in two GC cells. Given that AMPK mediated the phosphorylation of HNF4α, and prevented transcription factor HNF4α from entering the nucleus to form dimer and DNA binding to improve the degradation of HNF4α (Leclerc et al., 2001). We also observed that BBR (20 μM) upregulated the protein level of phosphor-AMPK (p-AMPK) and BBR (30 μM) upregulated the mRNA level of total AMPK in GC cells,MET increased the mRNA expression of AMPK in GC cells, although no statistically significant differences were found in the total protein level of AMPK after BBR and MET treatment. To further confirm the AMPK may be the potential upstream of HNF4α in GC cells, we used Compound C (AMPK-kinase inhibitor) and BBR to treat two GC cells. We found that BBR downregulated the protein level of HNF4α and Compound C upregulated the expression of HNF4α, Compound C reversed the effect of BBR on the HNF4α, suggesting inhibition of HNF4α by BBR was AMPK dependent. There was a negative correlation between p-AMPK and HNF4α. That means, BBR upregulated the protein of p-AMPK and Compound C downregulated the expression. This phenomenon indicated that HNF4α may be the downstream of AMPK and BBR could regulate the AMPK/HNF4α pathway in gastric cancer cells.

WNT/β-catenin signaling is important for cancer progression, including tumor initiation, tumor growth, cell senescence, cell death, differentiation and metastasis (Anastas and Moon, 2013). It was also reported that HNF4α-WNT5A regulation was the cross-talk between the AMPK metabolic pathway and the WNT signaling pathway (Nam et al., 2014). WNT5A, a member of the WNT family, played an important role in gastric cancer invasion and metastasis (Kanzawa et al., 2013; Ara et al., 2016). Knockdown of WNT5A reduced the metastasis ability of gastric cancer cells (Hanaki et al., 2012). Then, we explored how BBR acted on WNT/β-catenin signaling pathway in GC cell lines. Our study demonstrated that BBR downregulated the protein and mRNA levels of WNT5A and cytoplasmic β-catenin in AGS and SGC7901 cell lines. To further identify the relationship between HNF4α and WNT signal pathway. SiRNA- HNF4α SGC7901 cell model was used to detect the expression of WNT pathway. The results showed that knockdown of HNF4α decreased significantly the protein and mRNA expression levels of WNT5A, cytoplasmic β-catenin and cyclinD1 *in vitro*, and increased significantly the expression of E-cadherin comparing with control cells. HNF4α was reported to compete with β-catenin for binding to TCF4, regulating Epithelial-to-mesenchymal transition (EMT) (Yao et al., 2016). Loss of E-cadherin is regarded as a major conventional marker of the EMT (Alotaibi et al., 2015; Yamashita et al., 2018). Our study validated that WNT5A was a downstream target gene of HNF4α and showed that knockdown of HNF4α decreased the expression of WNT5A and cytoplasmic β-catenin in GC cell line, which is consistent with the study of Chang et al. (2015). But studies have illustrated, in small intestine and colon cancer, loss of HNF4α activated the WNT/β-catenin signaling pathway. In these cancer tissues, HNF4α was low expressed compared with adjacent normal tissues (Cattin et al., 2009; Yao et al., 2016).

Meanwhile, we analyzed that effect of BBR and HNF4α on gastric cancer tumor *in vivo*. Our results showed that BBR and knockdown of HNF4α suppressed the growth of SGC7901 in nude mice, HNF4α played a critical role in gastric cancer development and might serve as a potential therapy biomarker. Consistent with *in vitro* experiments, BBR downregulated HNF4α, WNT5A, and cytoplasmic β-catenin expression in somatic tumor tissues, the same effect as knocking out HNF4α gene.

Taken together, in the present study, we demonstrated that BBR, an anti-diabetes herb monomer, not only had proliferation inhibition effect on GC cell lines through downregulating cell cycle protein CyclinD1, attenuated the invasion and migration of GC cells by decreasing MMP-3, but also suppressed the growth of gastric tumor. The anti-gastric cancer mechanism of BBR might be involved in AMPK-HNF4α-WNT5A signaling pathway. BBR could modulate HNF4α through AMPK signaling. Moreover, HNF4α could regulate WNT-β-catenin-E-cadherin signaling to attenuate the growth, invasion and metastasis of gastric cancer. Therefore, HNF4α, a nuclear receptor that activated the expression of genes involved in glucose, fatty acid and cholesterol metabolism, was also involved in the development of gastric cancer. HNF4α antagonists, such as BBR, could be a promising anti-gastric cancer treatment supplement.

## Ethics Statement

The study was carried out in a accordance with the recommendations of Institutional Animal Care and Use Committee at Tongji Medical College (Huazhong University of Science and Technology ACUC No. S787). The study protocol was approved by Institutional Animal Care and Use Committee, Tongji Medical College, Huazhong University of Science and Technology.

## Author Contributions

PY designed the study. QH, LL, XZ, and LX conducted the experiments. QH wrote and revised the manuscript. All authors approved the final version to be published.

## Conflict of Interest Statement

The authors declare that the research was conducted in the absence of any commercial or financial relationships that could be construed as a potential conflict of interest.

## Abbreviations

AICAR: 5-Aminoimidazole-4-carboxamide1-β-D-ribofurano side
AMPK: adenosine 5′-monophosphate (AMP)-activated protein kinase
BBR: berberine
GC: gastric cancer
GFP: green fluorescent protein
HNF4α: hepatocyte nuclear factor-4α
MET: metformin.

## References

[B1] Alotaibi H. Basilicata M. F. Shehwana H. Kosowan T. Schreck I. Braeutigam C. (2015). Enhancer cooperativity as a novel mechanism underlying the transcriptional regulation of E-cadherin during mesenchymal to epithelial transition. *Biochim. Biophys. Acta* 1849 731–742. 10.1016/j.bbagrm.2015.01.005 25652130

[B2] Anastas J. N. Moon R. T. (2013). WNT signalling pathways as therapeutic targets in cancer. *Nat. Rev. Cancer* 13 11–26. 10.1038/nrc3419 23258168

[B3] Ara H. Takagishi M. Enomoto A. Asai M. Ushida K. Asai N. (2016). Role for Daple in non-canonical Wnt signaling during gastric cancer invasion and metastasis. *Cancer Sci.* 107 133–139. 10.1111/cas.12848 26577606 PMC4768387

[B4] Balakrishnan M. George R. Sharma A. Graham D. Y. (2017). Changing trends in stomach cancer throughout the world. *Curr. Gastroenterol. Rep.* 19 36. 10.1007/s11894-017-0575-8 28730504 PMC6918953

[B5] Cattin A. L. Le Beyec J. Barreau F. Saint-Just S. Houllier A. Gonzalez F. J. (2009). Hepatocyte nuclear factor 4 α, a key factor for homeostasis, cell architecture, and barrier function of the adult intestinal epithelium. *Mol. Cell. Biol.* 29 6294–6308. 10.1128/MCB.00939-09 19805521 PMC2786690

[B6] Chang H. R. Nam S. Kook M. Kim K. Liu X. Yao H. (2015). HNF4α is a therapeutic target that links AMPK to WNT signaling in early-stage gastric cancer. *Gut* 65 19–32. 10.1136/gutjnl-2014-307918 25410163 PMC4717359

[B7] Chen W. Sun K. Zheng R. Zeng H. Zhang S. Xia C. (2018). Cancer incidence and mortality in China. *Chin. J. Cancer Res.* 30 1–12. 10.21147/j.issn.1000-9604.2018.01.01 29545714 PMC5842223

[B8] Chu C. Liu X. Bai X. Zhao T. Wang M. Xu R. (2018). MiR-519d suppresses breast cancer tumorigenesis and metastasis via targeting MMP3. *Int. J. Biol. Sci.* 14 228–236. 10.7150/ijbs.22849 29483840 PMC5821043

[B9] Courtois S. Durán R. V. Giraud J. Sifré E. Izotte J. Mégraud F. (2017). Metformin targets gastric cancer stem cells. *Eur. J. Cancer* 84 193–201. 10.1016/j.ejca.2017.07.020 28822889

[B10] Dang C. V. (2012). MYC on the path to cancer. *Cell* 149 22–35. 10.1016/j.cell.2012.03.003 22464321 PMC3345192

[B11] Darsigny M. Babeu J. P. Seidman E. G. Gendron F. P. Levy E. Carrier J. (2010). Hepatocyte nuclear factor-4 αpromotes gut neoplasia in mice and protects against the production of reactive oxygen species. *Cancer Res.* 70 9423–9433. 10.1158/0008-5472.CAN-10-1697 21062980

[B12] Daugan M. Dufaÿ Wojcicki A. d’Hayer B. Boudy V. (2016). Metformin: an anti-diabetic drug to fight cancer. *Pharmacol. Res.* 113 675–685. 10.1016/j.phrs.2016.10.006 27720766

[B13] Deng J. Y. Liang H. (2014). Clinical significance of lymph node metastasis in gastric cancer. *World J. Gastroenterol.* 20 3967–3975. 10.3748/wjg.v20.i14.3967 24744586 PMC3983452

[B14] Gao Z. Leng S. Lu F. Xie M. J. Xu L. J. Wang K. F. (2008). Effect of berberine on expression of hepatocyte nuclear factor-4alpha in rats with fructose-induced insulin resistance. *J. Huazhong Univ. Sci. Technol.* 28 261–265. 10.1007/s11596-008-0307-2 18563319

[B15] Gérard C. Goldbeter A. (2014). The balance between cell cycle arrest and cell proliferation: control by the extracellular matrix and by contact inhibition. *Interface focus* 4:20130075. 10.1098/rsfs.2013.0075 24904738 PMC3996587

[B16] Gong J. Hu M. Huang Z. Fang K. Wang D. K. Chen Q. (2017). Berberine attenuates intestinal mucosal barrier dysfunction in type 2 diabetic rats. *Front. Pharmacol.* 8:42. 10.3389/fphar.2017.00042 28217099 PMC5290458

[B17] Hanaki H. Yamamoto H. Sakane H. Matsumoto S. Ohdan H. Sato A. (2012). An anti-wnt5a antibody suppresses metastasis of gastric cancer cells *in vivo* by inhibiting receptor-mediated endocytosis. *Mol. Cancer Ther.* 11 298–307. 10.1158/1535-7163.MCT-11-0682 22101459

[B18] He Y. Tan X. Hu H. Wang Q. Hu X. Cai X. (2018). Metformin inhibits the migration and invasion of esophageal squamous cell carcinoma cells by downregulating the protein kinase B signaling pathway. *Oncol. Lett.* 15 2939–2945. 10.3892/ol.2017.7699 29435022 PMC5778829

[B19] Huang J. F. Du W. X. Chen J. J. (2016). Elevated expression of matrix metalloproteinase-3 in human osteosarcoma and its association with tumor metastasis. *J. BUON* 21 1279–1286. 27837634

[B20] Jucá P. C. F. C. Corrêa S. Vignal G. M. Accioly M. T. S. Lustosa S. A. S. Abdelhay E. (2017). HNF4A expression as a potential diagnostic tool to discriminate primary gastric cancer from breast cancer metastasis in a Brazilian cohort. *Diagn. Pathol.* 12:43. 10.1186/s13000-017-0635-2 28583188 PMC5460322

[B21] Kanzawa M. Semba S. Hara S. Itoh T. Yokozaki H. (2013). WNT5A is a key regulator of the epithelial-mesenchymal transition and cancer stem cell properties in human gastric carcinoma cells. *Pathobiology* 80 235–244. 10.1159/000346843 23615002

[B22] Kato Y. Maeda T. Suzuki A. Baba Y. (2018). Cancer metabolism: new insights into classic characteristics. *Jpn. Dent. Sci. Rev.* 54 8–21. 10.1016/j.jdsr.2017.08.003 29628997 PMC5884251

[B23] Kobayashi P. E. Fonseca-Alves C. E. Rivera-Calderón L. G. Carvalho M. Kuasne H. Rogatto S. R. (2018). Deregulation of E-cadherin, β-catenin, APC and Caveolin-1 expression occurs in canine prostate cancer and metastatic processes. *Res. Vet. Sci.* 118 254–261. 10.1016/j.rvsc.2018.03.004 29529534

[B24] Kumar A. Ekavali Chopra K. Mukherjee M. Pottabathini R. Dhull D. K. (2015). Current knowledge and pharmacological profile of berberine: an update. *Eur. J. Pharmacol.* 761 288–297. 10.1016/j.ejphar.2015.05.068 26092760

[B25] Larue L. Delmas V. (2006). The WNT/Beta-catenin pathway in melanoma. *Front. Biosci.* 11 733–742. 10.2741/1831 16146765

[B26] Leclerc I. Lenzner C. Gourdon L. Vaulont S. Kahn A. Viollet B. (2001). Hepatocyte nuclear factor-4alpha involved in type 1 maturity-onset diabetes of the young is a novel target of AMP-activated protein kinase. *Diabetes Metab. Res. Rev.* 50 1515–1521. 11423471 10.2337/diabetes.50.7.1515

[B27] Leng S. H. Lu F. E. Xu L. J. (2004). Therapeutic effects of berberine in impaired glucose tolerance rats and its influence on insulin secretion. *Acta Pharmacol. Sin.* 25 496–502. 15066220

[B28] Li H. L. Wu H. Zhang B. B. Shi H. L. Wu X. J. (2016). MAPK pathways are involved in the inhibitory effect of berberine hydrochloride on gastric cancer MGC 803 cell proliferation and IL-8 secretion *in vitro* and *in vivo*. *Mol. Med. Rep.* 14 1430–1438. 10.3892/mmr.2016.5361 27278862

[B29] Li P. Zhang C. Gao P. Chen X. W. Ma B. Yu D. (2018). Metformin use and its effect on gastric cancer in patients with type 2 diabetes: a systematic review of observational studies. *Oncol. Lett.* 15 1191–1199. 10.3892/ol.2017.7370 29391902 PMC5769385

[B30] Love-Gregory L. Permutt M. A. (2007). HNF4A genetic variants: role in diabetes. *Curr. Opin. Clin. Nutr. Metab. Care* 10 397–402. 10.1097/MCO.0b013e3281e3888d 17563455

[B31] Ma Y. Wei X. Wu Z. J. (2017). HNF-4α promotes multidrug resistance of gastric cancer cells through the modulation of cell apoptosis. *Oncol. Lett.* 14 6477–6484. 10.3892/ol.2017.7095 29344114 PMC5754880

[B32] Mao L. Chen Q. Gong K. Xu X. L. Xie Y. R. Zhang W. Q. (2018). Berberine decelerates glucose metabolism via suppression of mTOR-dependent HIF-1α protein synthesis in colon cancer cells. *Oncol. Rep.* 39 2436–2442. 10.3892/or.2018.6318 29565467

[B33] Nam S. Chang H. R. Kim K. T. Kook M. C. Hong D. Kwon C. H. (2014). PATHOME: an algorithm for accurately detecting differentially expressed subpathways. *Oncogen* 33 4941–4951. 10.1038/onc.2014.80 24681952 PMC4182295

[B34] Pirillo A. Catapano A. L. (2015). Berberine, a plant alkaloid with lipid- and glucose-lowering properties: from *in vitro* evidence to clinical studies. *Atherosclerosis* 243 449–461. 10.1016/j.atherosclerosis.2015.09.032 26520899

[B35] Rhee J. Ge H. Yang W. Fan M. Handschin C. Cooper M. (2006). Partnership of PGC-1alpha and HNF4alpha in the regulation of lipoprotein metabolism. *J. Biol. Chem.* 281 14683–14690. 10.1074/jbc.M512636200 16574644

[B36] Simi A. K. Anlas A. A. Stallings-Mann M. Zhang S. Hsia T. Cichon M. A. (2018). A soft microenvironment protects from failure of midbody abscission and multinucleation downstream of the EMT-promoting transcription factor Snail. *Cancer Res.* 78 2277–2289. 10.1158/0008-5472.CAN-17-2899 29483094 PMC5932229

[B37] Song I. S. Han J. Lee H. K. (2015). Metformin as an anticancer drug: a commentary on the metabolic determinants of cancer cell sensitivity to glucose limitation and biguanides. *J. Diabetes Investig.* 6 516–518. 10.1111/jdi.12300 26421142 PMC4578488

[B38] Tabeshpour J. Imenshahidi M. Hosseinzadeh H. (2017). A review of the effects of Berberis vulgaris and its major component, berberine, in metabolic syndrome. *Iran. J. Basic Med. Sci.* 20 557–568. 10.22038/IJBMS.2017.8682 28656091 PMC5478784

[B39] Torre L. A. Siegel R. L. Ward E. M. Jemal A. (2016). Global cancer incidence and mortality rates and trends–an update. *Cancer Epidemiol. Biomarkers* 25 16–27. 10.1158/1055-9965.EPI-15-0578 26667886

[B40] Tseng C. H. Tseng F. H. (2014). Diabetes and gastric cancer: the potential links. *World J Gastroenterol.* 20 701–1711. 10.3748/wjg.v20.i7.1701 24587649 PMC3930970

[B41] Valaee S. Yaghoobi M. M. Shamsara M. (2017). Metformin inhibits gastric cancer cells metastatic traits through suppression of epithelial-mesenchymal transition in a glucose-independent manner. *PLoS One* 12:e0174486. 10.1371/journal.pone.0174486 28334027 PMC5363973

[B42] Van der Post R. S. Bult P. Vogelaar I. P. Ligtenberg M. J. L. Hoogerbrugge N. van Krieken J. H. (2014). HNF4A immunohistochemistry facilitates distinction between primary and metastatic breast and gastric carcinoma. *Virchows Arch.* 464 673–679. 10.1007/s00428-014-1574-x 24711169

[B43] Wang H. Zhu C. Ying Y. Luo L. Huang D. Luo Z. (2018). Metformin and berberine, two versatile drugs in treatment of common metabolic diseases. *Oncotarget* 9 10135–10146. 10.18632/oncotarget.20807 29515798 PMC5839379

[B44] Wang J. Yang S. Cai X. Dong J. Chen Z. Q. Wang R. (2016). Berberine inhibits EGFR signaling and enhances the antitumor effects of EGFR inhibitors in gastric cancer. *Oncotarget* 7 76076–76086. 10.18632/oncotarget.12589 27738318 PMC5342797

[B45] Wang Q. He G. Hou M. Chen L. T. Chen S. W. Xu A. (2018). Cell cycle regulation by alternative polyadenylation of CCND1. *Sci. Rep.* 8:6824. 10.1038/s41598-018-25141-0 29717174 PMC5931507

[B46] Wang Z. Q. Lu F. E. Leng S. H. Fang X. S. Chen G. Wang Z. S. (2008). Facilitating effects of berberine on rat pancreatic islets through modulating hepatic nuclear factor 4 alpha expression and glucokinase activity. *World J. Gastroenterol.* 14 6004–6011. 10.3748/wjg.14.6004 18932278 PMC2760199

[B47] Wei S. Zhang M. Yu Y. Lan X. Yao F. Yan X. (2016). Berberine attenuates development of the hepatic gluconeogenesis and lipid metabolism disorder in type 2 diabetic mice and in palmitate-incubated HepG2 cells through suppression of the HNF-4α miR122 pathway. *PLoS One* 11:e0152097. 10.1371/journal.pone.0152097 27011261 PMC4806913

[B48] Wei W. Zhao H. Wang A. Sui M. Liang K. Deng H. Y. (2012). A clinical study on the short-term effect of berberine in comparison to metformin on the metabolic characteristics of women with polycystic ovary syndrome. *Eur. J. Endocrinol.* 166 99–105. 10.1530/EJE-11-0616 22019891

[B49] Xiang X. Zhao X. Qu H. Li D. Yang D. Pu J. (2015). Hepatocyte nuclear factor 4 alpha promotes the invasion, metastasis and angiogenesis of neuroblastoma cells via targeting matrix metalloproteinase 14. *Cancer Lett.* 359 187–197. 10.1016/j.canlet.2015.01.008 25592038

[B50] Xu J. Changyong E. Yao Y. Ren S. Wang G. Jin H. (2016). Matrix metalloproteinase expression and molecular interaction network analysis in gastric cancer. *Oncol. Lett.* 12 2403–2408. 10.3892/ol.2016.5013 27698806 PMC5038516

[B51] Yamashita N. Tokunaga E. Iimori M. Inoue Y. Tanaka K. Kitao H. (2018). Epithelial paradox: clinical significance of coexpression of e-cadherin and vimentin with regard to invasion and metastasis of breast cancer. *Clin. Breast Cancer* 18 e1003–e1009. 10.1016/j.clbc.2018.02.002 29526677

[B52] Yan Z. Q. Leng S. H. Lu F. E. Lu X. H. Dong H. Gao Z. Q. (2008). [Effects of berberine on expression of hepatocyte nuclear factor 4alpha and glucokinase activity in mouse primary hepatocytes]. *Zhongguo Zhong yao za zhi Zhongguo zhongyao zazh China J. Chin. Mat. Med.* 33 2105–2109. 19160796

[B53] Yang Y. Zhang N. Li K. Chen J. Qiu L. Zhang J. F. (2018). Integration of microRNA-mRNA profiles and pathway analysis of plant isoquinoline alkaloid berberine in SGC-7901 gastric cancers cells. *Drug Des. Dev. Ther.* 12 393–408. 10.2147/DDDT.S155993 29535501 PMC5836656

[B54] Yao H. S. Wang J. Zhang X. P. Wang L. Z. Wang Y. Li X. X. (2016). Hepatocyte nuclear factor 4α suppresses the aggravation of colon carcinoma. *Mol. Carcinogens* 55 458–472. 10.1002/mc.22294 25808746

[B55] Yi P. Lu F. E. Xu L. J. Chen G. Dong H. Wang K. F. (2008). Berberine reverses free-fatty-acid-induced insulin resistance in 3T3-L1 adipocytes through targeting IKKbeta. *World J. Gastroenterol.* 14 876–883. 10.3748/wjg.14.876 18240344 PMC2687054

[B56] Zhang X. Z. Wang L. Liu D. W. Tang G. Y. Zhang H. Y. (2014). Synergistic inhibitory effect of berberine and d-limonene on human gastric carcinoma cell line MGC803. *J. Med. Food* 17 955–962. 10.1089/jmf.2013.2967 25045784 PMC4152785

[B57] Zhou X. L. Xue W. H. Ding X. F. Li L. F. Dou M. M. Zhang W. J. (2017). Association between metformin and the risk of gastric cancer in patients with type 2 diabetes mellitus: a meta-analysis of cohort studies. *Oncotarget* 8 55622–55631. 10.18632/oncotarget.16973 28903449 PMC5589688

[B58] Zhu T. Hu X. Wei P. Shan G. Z. (2018). Molecular background of the regional lymph node metastasis of gastric cancer. *Oncol. Lett.* 15 3409–3414. 10.3892/ol.2018.7813 29556271 PMC5844018

